# Vitamin B6 Inhibits High Glucose-Induced Islet β Cell Apoptosis by Upregulating Autophagy

**DOI:** 10.3390/metabo12111048

**Published:** 2022-10-31

**Authors:** Yu Zhang, Xi-an Zhou, Chuxin Liu, Qingwu Shen, Yanyang Wu

**Affiliations:** 1 Key Laboratory for Food Science and Biotechnology of Hunan Province, College of Food Science and Technology, Hunan Agricultural University, Changsha 410128, China; 2 Horticulture and Landscape College, Hunan Agricultural University, Changsha 410128, China; 3 Hunan Co-Innovation Center for Utilization of Botanical Functional Ingredients, Changsha 410128, China; 4 State Key Laboratory of Subhealth Intervention Technology, Changsha 410128, China

**Keywords:** egg, diabetes, vitamin B6, islet β cells

## Abstract

Vitamin B6 may alleviate diabetes by regulating insulin secretion and increasing insulin sensitivity, but its mechanism remains to be explored. In this study, vitamin B6-mediated autophagy and high glucose-induced apoptosis were tested to investigate the mechanism by which vitamin B6 regulates insulin release. The results showed that 20 mM glucose increased the apoptosis rate from 10.39% to 22.44%. Vitamin B6 reduced the apoptosis rate of RIN-m5F cells from 22.44% to 11.31%. Our data also showed that the vitamin B6 content in processed eggs was decreased and that the hydrothermal process did not affect the bioactivity of vitamin B6. Vitamin B6 increased the number of autophagosomes and the ratio of autophagosome marker protein microtubule associated protein 1 light chain 3 beta to microtubule associated protein 1 light chain 3 alpha (LC3-II/LC3-I). It also decreased the amount of sequetosome 1 (SQSTM1/p62) and inhibited the phosphorylation of p70 ribosomal protein S6 kinase (p70S6K) under normal and high glucose stress. Another study showed that vitamin B6 inhibited the apoptosis rate, whereas the autophagy inhibitor 3-methyladenine (3-MA) blocked the protective effect of vitamin B6 against apoptosis induced by high glucose. The hydrothermal process decreased the vitamin B6 content in eggs but had no effect on the cytoprotective function of vitamin B6 in RIN-m5f cells. In conclusion, we demonstrated that vitamin B6-mediated autophagy protected RIN-m5f cells from high glucose-induced apoptosis might via the mTOR-dependent pathway. Our data also suggest that low temperatures and short-term hydrothermal processes are beneficial for dietary eggs.

## 1. Introduction

Vitamin B6 is an essential nutrient that can promote health and growth. There are many different forms of vitamin B6, including pyridoxine (PN), pyridoxal (PL), pyridoxamine (PM), and the 5′-phosphate derivatives pyridoxine 5′-phosphate (PNP), pyridoxal 5′-phosphate (PLP), and pyridoxamine phosphate (PMP) [1]. Vitamin B6 can scavenge oxygen-active substances and inhibit the formation of genotoxic compounds associated with advanced glycation end products, ageing, and diabetes [2]. Vitamin B6 has been shown to effectively alleviate diabetes and its complications [1,2,3]. Vitamin B6 deficiency promoted the development of complications and the onset of diabetes, suggesting that vitamin B6 deficiency may be both a consequence of diabetes and a cause of diabetes [4]. Blood levels of vitamin B6 appear to be negatively correlated with the development of diabetes [4,5]. Eggs are among foods consumed by humans on a daily basis. They are rich in nutrients, such as proteins and vitamins. Hydrothermal processing is a common method of producing dietary eggs. However, its effects on the content and biological activity of vitamins in eggs have yet to be studied.

Diabetes is a metabolic disease characterized by hyperglycaemia. Diabetes is divided into type I diabetes (T1DM), type II diabetes (T2DM), and gestational diabetes [6]. Gestational diabetes is a type of diabetes that occurs or is diagnosed during pregnancy [7]. T1DM is called insulin-dependent diabetes. It is characterized by hyperglycaemia caused by absolute insulin deficiency [8]. This disease is mainly due to immune-mediated damage to pancreatic β-cells [8]. T2DM, known as noninsulin-dependent diabetes, is a chronic metabolic disease. It is characterized by islet β-cell damage, high insulin deficiency, or insulin resistance [9]. Autophagy plays many important roles in β-cell function and promotes survival [10].

Autophagy is a process of the degradation of intracellular substances [11]. It maintains the number, structure, and function of islet β-cells and eliminates dysfunctional mitochondria in insulinoma cells. Exenatide and liraglutide mediate the secretion of insulin by promoting autophagy [12,13]. Homeostasis of islet β-cells is inextricably linked to autophagy [14]. Once autophagy is disrupted, islet β-cells lose cellular functions, as indicated by increased apoptosis and decreased proliferation of β-cells, resulting in a decrease in β-cell mass [15]. 

The role of autophagy in cell death is complex. Some reports have stated that autophagy promoted cell death. Thus, autophagic cell death was induced after the inhibition of caspase. Autophagy triggers the maturation of cathepsin D (CTSD) to promote apoptosis [16]. Autophagy can also trigger cytoprotection. Thus, autophagy regulates the removal of protein aggregates [17]. It also inhibits the activity of the cyclin-dependent kinase inhibitor p27 and promotes survival [18]. It also removes damaged organelles, such as mitochondria, lysosomes, or the endoplasmic reticulum, to prevent apoptosis [19].

Currently, regulation of autophagy by natural products and a healthy diet is an effective method to alleviate diabetes and its complications [20,21]. In this study, we aimed to evaluate the mechanism of vitamin B6-mediated cell death via the induction of autophagy in β-cells and to investigate the effects of the hydrothermal process on the concentration and bioactivity of vitamin B6 in eggs.

## 2. Materials and Methods

### 2.1. Reagents and Antibodies

Vitamin B6 (8030) was purchased from Beijing Solarbio Biological Technology Co., Ltd. (Shanghai, China). Anti-LC3 polyclonal antibody (PM036), anti-LC3 monoclonal antibody (M186-3), and anti-SQSTM1/p62 antibody (PM045) were purchased from Medical Biological Laboratory (MBL, Tokyo, Japan). Anti-p70S6K antibody (2708) and anti-phosphorylated p70S6K antibody (9206) were purchased from Cell Signaling Technology (Beverly, MA, USA). Anti-3-phosphoglyceraldehyde dehydrogenase (GAPDH) antibody (ZB002) was purchased from YTHX Biotechnology Co., Ltd. (Beijing, China). Propidium iodide (PI) and annexin V fluorescein isothiocyanate (FITC) for flow cytometry were purchased from BD Biotechnology Research Co., Ltd. (California, CA, USA) and goat anti-mouse IgG (1070-05) and goat anti-rabbit antibodies (4050-05) were purchased from Southern Biotechnology Company (Birmingham, UK). DQ-red BSA (D-12051) and Alexa Fluor 488 goat anti-rabbit antibody (A11034) were purchased from Invitrogen Life Technology (Shanghai, China). Pyridoxine hydrochloride (≥99%), pyridoxal hydrochloride (≥99%), pyridoxamine dihydrochloride (≥99%), sodium octane sulfonate (98%), and triethylamine (chromatographic purity) were all purchased from Beijing Xuejiete Technology Co., Ltd. (Beijing, China), and sodium hydroxide (analytical purity), glacial acetic acid (analytical purity), hydrochloric acid (analytical purity), and methanol (chromatographic purity) were purchased from Sinopharm Chemical Reagent Co., Ltd. (Shanghai, China).

### 2.2. Cell Culture

RIN-m5F cells were cultured in Roswell Park Memorial Institute (RPMI) 1640 medium with 10% fetal bovine serum (FBS) (BI, Herzliya, Israel) at 37 °C and 5% CO_2_.

### 2.3. Immunofluorescence Staining

RIN-m5F cells were cultured in 24-well plates with round cover glasses. The cells were washed with phosphate buffered saline (PBS) three times and then fixed with 4% paraformaldehyde for 10 min. The cells were sealed with PBS containing 10% FBS for 30 min after washing three times with PBS. The cells were washed with PBS three times after incubation with anti-LC3 antibody at 37 °C for 1 h. The cells were then incubated with Alexa Fluor 488 goat anti-rabbit antibody at 37 °C for 1 h and finally sealed with a fluorescence quencher. Images were observed with a confocal microscope (Zeiss LSM 710, Jena, Germany).

### 2.4. Western Blotting

RIN-m5F cells were cultured in 6-well plates. The medium was then removed, and the cells were washed with PBS three times. The cells were treated with 2% sodium dodecyl sulfate (SDS, 200 μL per well). The extract was then heated at 100 °C for 10 min and mixed with 6× protein loading buffer (J21020, Transgen, Beijing, China). The proteins in this extract were separated by sodium dodecyl sulfate–polyacrylamide gel electrophoresis (SDS–PAGE) after the extract was heated for 10 min at 100 °C (4% stacked gel, 15% separated gel, 50 V for 30 min, 90 V for 120 min) and then transferred to nitrocellulose membranes (250 mA, 30–60 min). The membranes were blocked with 5% skimmed milk powder for 1 h and incubated with primary antibody at 4 °C overnight. The membranes were incubated with secondary antibody for 1 h after washing with PBST (PBS plus 0.2% Tween-20) three times. The membranes were then imaged with a luminescent image analyzer (Model: Image Quant LAS4000 Mini, Serial No. 3614294; GE Healthcare Bio-Sciences AB, Uppsala, Sweden) after incubation with WesternBrightTM ECL chemiluminescent HRP substrate (SuperSignal West Dura, 32106, Thermo Pierce) for a few minutes.

### 2.5. Cell Apoptosis Assay

A PI-Annexin V-FITC Apoptosis Detection Kit was used for flow cytometry analysis of apoptotic cells. Rin-m5F cells were cultured in 12-well plates for 24 or 36 h, washed with PBS, and digested with trypsin. The cells were obtained by centrifugation and suspended in 100 μL binding buffer (1 × 10^6^ cells/mL). Each tube was stained with 5 μL annexin V-FITC and 5 μL PI for 15 min, and then 400 μL binding buffer was added. The fluorescence intensity of these cells was analyzed by flow cytometry (Beckman MoFlo XDP, Georgia, GA, USA).

### 2.6. Testing the Viability of RIN-m5F Cells

The cells were cultured in 96-well plates at a density of 10,000 cells per well for 24 h. Each well was supplemented with the substances, and the plates were incubated at 37 °C for an appropriate time. Then, 10 μL Cell Counting Kit-8 (CCK8) solution was added to each well, and the plates were incubated at 37 °C for 1–4 h. The absorbance at 450 nm was measured using a microplate reader.

### 2.7. Test of Vitamin B6 Content

The concentration of vitamin B6 was determined by high-performance liquid chromatography (HPLC) (Shimadzu LC-20A, Kyoto, Japan) using a C18 chromatographic column with a column length of 150 mm, column inner diameter of 4.6 mm, and column filler particle size of 5 μm. Fifty milliliters of methanol, 2.0 g of sodium octane sulfonate, and 2.5 mL of triethylamine were dissolved in water to reach a final volume of 1000 mL, and this solution was used as the mobile phase. The detection excitation wavelength was 293 nm, and the emission wavelength was 395 nm.

## 3. Results

### 3.1. Vitamin B6 Inhibited Apoptosis Induced by High Glucose

**Figure 1:**
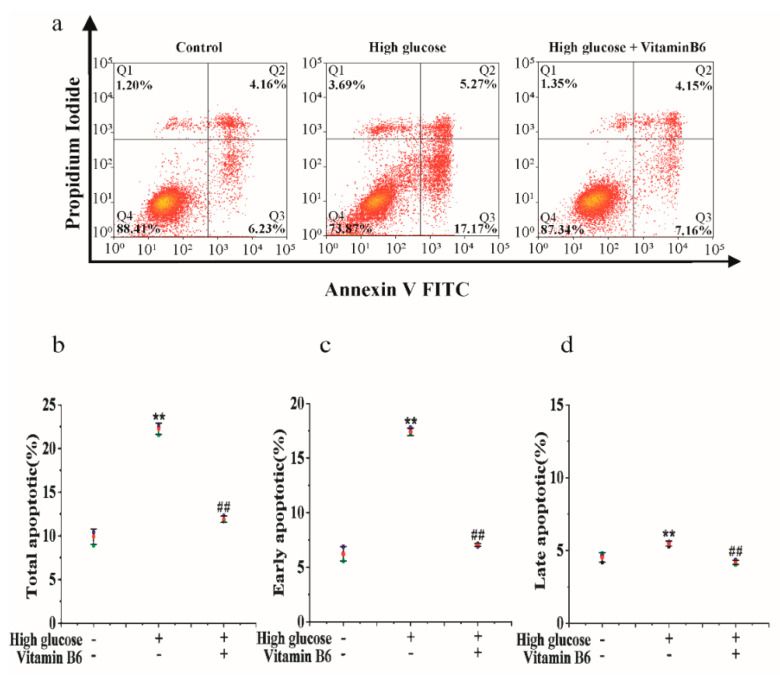
Vitamin B6 protected cells from apoptosis induced by high glucose. (**a**) Apoptosis rates of RIN-m5F cells cultured with or without 20 μM glucose or with 20 μM vitamin B6 plus 20 μM glucose for 36 h were measured by flow cytometry. (**b**–**d**) Cells were treated as described in (**a**), and total apoptosis, late apoptosis, and early apoptosis were analyzed. The experiment was repeated at least three times (Error bars, s.d ** *p* < 0.01 indicates significant differences compared with the control group by one-way ANOVA. ## *p* < 0.01 indicates significant differences compared to the high glucose-treated group by one-way ANOVA.

To investigate the effect of vitamin B6 on apoptosis induced by high glucose, cells were treated with vitamin B6 under high glucose stress, and the apoptosis rate was tested by flow cytometry. The data showed that 20 mM glucose increased the apoptosis rate from 10.39% to 22.44%. Vitamin B6 reduced the apoptosis rate of RIN-m5f cells from 22.44% to 11.31% (Figure 1).

### 3.2. Effect of Hydrothermal Processing on the Biological Activity of Vitamin B6 in Eggs

**Figure 2:**
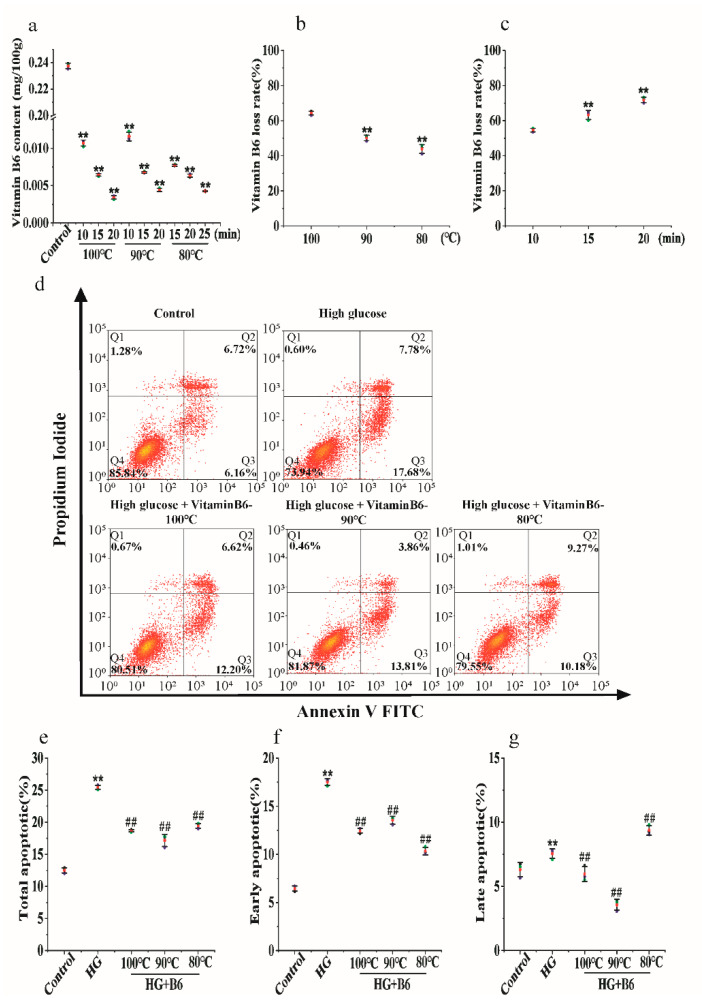
The effect of hydrothermal treatment on the concentration and biological activity of vitamin B6 in eggs. (**a**) Vitamin B6 content was determined after eggs were hydrothermally treated at 80, 90, or 100 °C for the indicated times. (**b**) Vitamin B6 content was determined after the eggs were hydrothermally annealed at 80, 90, or 100 °C for 10 min. (**c**) Vitamin B6 content was determined after eggs were hydrothermally annealed at 100 °C for the indicated times. (**d**) RIN-m5F cells were cultured with or without 20 μM glucose or with 20 μM hydrothermally prepared vitamin B6 plus 20 μM glucose for 36 h, and apoptosis rates were measured by flow cytometry. (**e**–**g**) Cells were treated as described in (**d**), and total apoptosis, late apoptosis, and early apoptosis rates were analyzed. The experiment was repeated at least three times (Error bars, s.d ** *p* < 0.01 indicates significant differences compared with the control group by one-way ANOVA. ## *p* < 0.01 indicates significant differences compared to the high glucose-treated group by one-way ANOVA.

Our results showed that the vitamin B6 content in eggs decreased from 0.0112 mg/100 g to 0.0030 mg/100 g after the eggs were hydrothermally processed at 100 °C for the indicated times. The content of vitamin B6 in eggs decreased from 0.0116 mg/100 g to 0.0043 mg/100 g after the eggs were hydrothermally processed at 90 °C for the indicated times. The change in vitamin B6 content in the 80 °C-treated group was the same as that in the 100 °C- and 90 °C-treated groups (Figure 2a). The vitamin B6 content was tested after the eggs were hydrothermally treated at 100 °C, 90 °C, and 80 °C for 15 min. Our results showed that the loss rate decreased from 65.26% to 44.66% with decreasing temperature (Figure 2b). At the same time, we boiled vitamin B6 at 100 °C for different times. Our results showed that the loss rate decreased from 54.56% to 72.19% with increasing time (Figure 2c). These data suggest that hydrothermal processing reduced the vitamin B6 content in eggs.

Vitamin B6 reduced the total apoptosis rate of RIN-m5F cells from 25.46% to 18.82%, 17.67%, or 19.45% (Figure 2e). Hydrothermal processing of vitamin B6 at 90 °C or 80 °C also reduced apoptosis induced by high glucose. There was no difference between the groups treated with processed vitamin B6 and those treated with unprocessed vitamin B6 (Figure 2d–g).

### 3.3. Vitamin B6 Induced Autophagy

**Figure 3:**
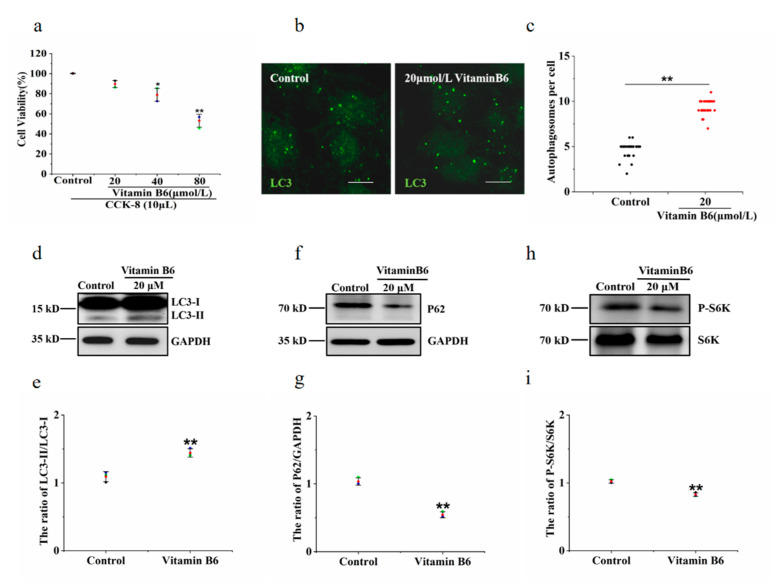
Vitamin B6 induced autophagy in RIN-m5F cells. (**a**) Cells were treated with the indicated concentration of vitamin B6, and cytotoxicity was assayed using a CCK-8 kit. Error bars, s.d. ** *p* < 0.01, * *p* < 0.05 indicates significant differences compared with the control group by one-way ANOVA. (**b**) RIN-m5F cells were treated with 20 μM vitamin B6 for 24 h and stained with anti-LC3 antibody (scale = 5 μm). (**c**) Cells were cultured as described in (**b**), and the number of autophagosomes per cell was calculated. At least 30 cells were counted by *t*-test analysis, and the experiment was repeated at least three times (Error bars, SD. ** *p* < 0.01 indicates significant differences compared with the control group). (**d**,**f**,**h**) RIN-m5F cells were treated with 20 μM vitamin B6, and Western blotting was performed to test the expression of autophagy-related proteins with anti-p62, anti-S6K, phospho-p70S6K, anti-LC3, and anti-GAPDH antibodies. (**e**,**g**,**i**) The LC3-II/LC3-I, P62/GAPDH and pS6K/S6K ratios in (**d**,**f**,**h**), respectively, were analyzed by integrated optical density (IOD) using Image-Pro Plus 6.0 software. (Error bars, SD. ** *p* < 0.01, * *p* < 0.05 indicates significant differences compared with the control group).

The cell survival rate of the group treated with 20 μM vitamin B6 showed no significant difference from the control group. In contrast, the cell survival rate was lower in the groups treated with 40 μM or 80 μM vitamin B6. Therefore, it can be concluded that 20 μM vitamin B6 had no effect on cell survival (Figure 3a). Microtubule-associated protein 1 light chain 3 (LC3) is a marker protein of autophagy. Cytoplasmic microtubule associated protein 1 light chain 3 alpha (LC3-I) is converted to microtubule associated protein 1 light chain 3 beta (LC3-II) and bound to the autophagic membrane when autophagy is promoted. The number of LC3-positive puncta and the LC3-II/LC3-I ratio were used to evaluate the degree of autophagy. To investigate the effects of vitamin B6 on autophagy, cells were treated with vitamin B6, and the number of autophagosomes was determined by immunofluorescence staining. The data showed that the number of autophagosomes was increased in the group treated with 20 μM vitamin B6 (Figure 3b,c). The LC3-II/LC3-I ratio was also significantly higher in the vitamin B6-treated group than in the control group (Figure 3d,e). Sequetosome 1 (p62) is a substrate protein that is specifically degraded during autophagy. Vitamin B6 significantly decreased the protein expression of p62, which is specifically degraded during autophagy (Figure 3f,g). mTOR is a negative regulator of autophagy [22]. The phosphorylation level of the substrate protein p70 ribosomal protein S6 kinase (p70S6K) may reflect the activity of mTOR, and the decrease in the phosphorylation level of p70S6K indicates that the activity of mTOR was inhibited [23]. Our results showed that vitamin B6 inhibited the phosphorylation of p70S6K (Figure 3h,i). From the above data, it can be concluded that vitamin B6 can induce autophagy under normal culture conditions.

### 3.4. Vitamin B6 Induced Autophagy under High Glucose Stress

**Figure 4:**
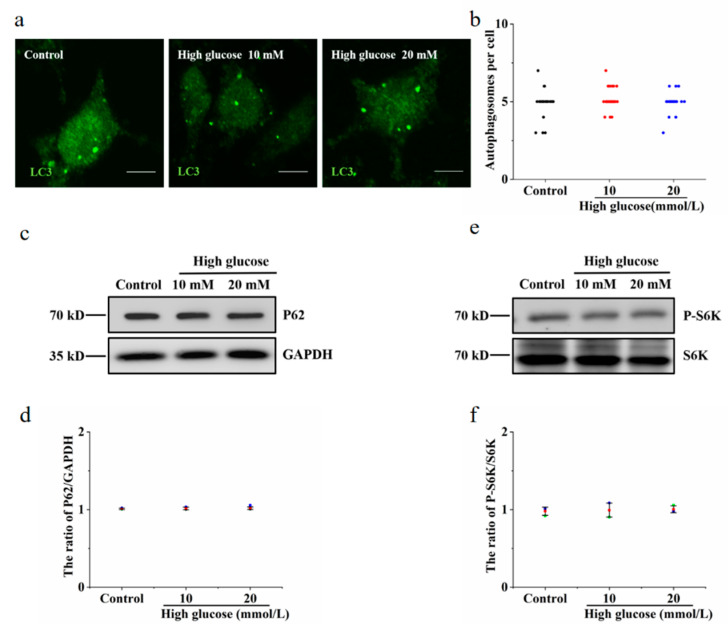
High glucose had no effect on autophagy in RIN-m5F cells. (**a**) Cells were cultured with 10 or 20 mM glucose for 36 h and stained with anti-LC3 antibody. (**b**) Cells were treated as described in (**a**), the number of autophagosomes per cell was calculated, and at least 30 cells were counted by *t*-test analysis. The experiment was repeated at least three times. (**c**,**e**) Cells were cultured as described in (**a**), and Western blotting was performed to test the expression of autophagy-related proteins with specific antibodies, such as anti-p62, anti-S6K, p70S6K, anti-LC3, and anti-GAPDH. (**d**,**f**) The P62/GAPDH and pS6K/S6K ratios in (**c**,**e**), respectively, were analyzed by IOD using Image-Pro Plus 6.0 software (Error bars, SD).

**Figure 5:**
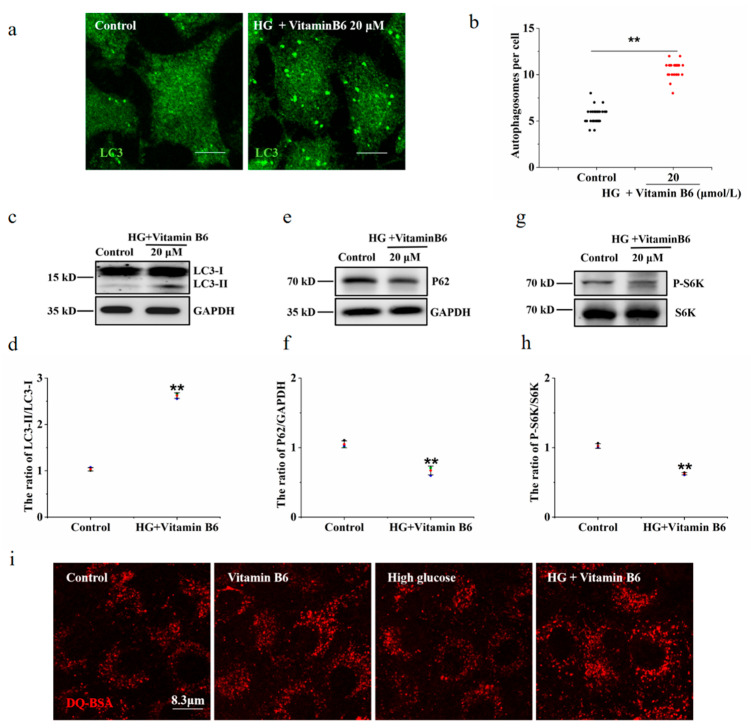
Vitamin B6 induced autophagy under high glucose stress. (**a**) Cells were treated with or without 20 μM vitamin B6 plus 20 mM glucose (HG) for 36 h, and the number of autophagosomes was assayed by immunofluorescence analysis. (**b**) Cells were treated as described in (**a**), mathematical statistics were performed to calculate the number of autophagosomes per cell, and at least 30 cells were counted by *t*-test analysis. The experiment was repeated at least three times. (**c**,**e**,**g**) Cells were treated as described in (**a**), and Western blotting was performed to test the expression of proteins with specific anti-p62, anti-S6K, anti-p70S6K, anti-LC3, and anti-GAPDH antibodies. (**d**,**f**,**h**) The ratio of LC3-II/LC3-I, P62/GAPDH, and pS6K/S6K ratios in (**c**,**e**,**g**), respectively, were analyzed by IOD using Image-Pro Plus 6.0 software (Error bars, SD. ** *p* < 0.01, indicates significant differences compared with the control group). (**i**) The cells were treated with or without glucose (HG), with vitamin B6 or with 20 μM glucose (HG) plus vitamin B6 for 36 h. The cells were then incubated with DQ-BSA for 3 h and imaged by confocal microscopy.

Hyperglycaemia is one of the clinical features of diabetes. To test the effect of vitamin B6 on autophagy under high glucose stress, RIN-m5F cells were treated with 10 mM and 20 mM glucose. The number of autophagosomes and the LC3-II/LC3-I ratio were observed. Our data showed that the number of autophagosomes was not changed in the group treated with 10 mM or 20 mM high glucose compared with the control group (Figure 4a,b). The expression of p62 protein and the phosphorylation level of p70S6K were not changed in the group treated with 10 mM or 20 mM high glucose (Figure 4c–f). The number of autophagosomes and the LC3-II/LC3-I ratio were increased in the group treated with vitamin B6 plus 20 mM high glucose (Figure 5a–d). The expression of p62 protein and the phosphorylation rate of p70S6K were also decreased (Figure 5e–h). To test the effect of vitamin B6 on lysosome degradation capacity, we labelled cells with DQ-BSA, a lysosome degradation indicator. As shown in Figure 5i, treatment with vitamin B6 or 20 mM high glucose plus vitamin B6 did not impair lysosome degradation activity. These data suggest that vitamin B6 induced autophagy under normal or high glucose stress.

### 3.5. 3-MA Inhibited Vitamin B6-Induced Autophagy under High Glucose Stress

**Figure 6:**
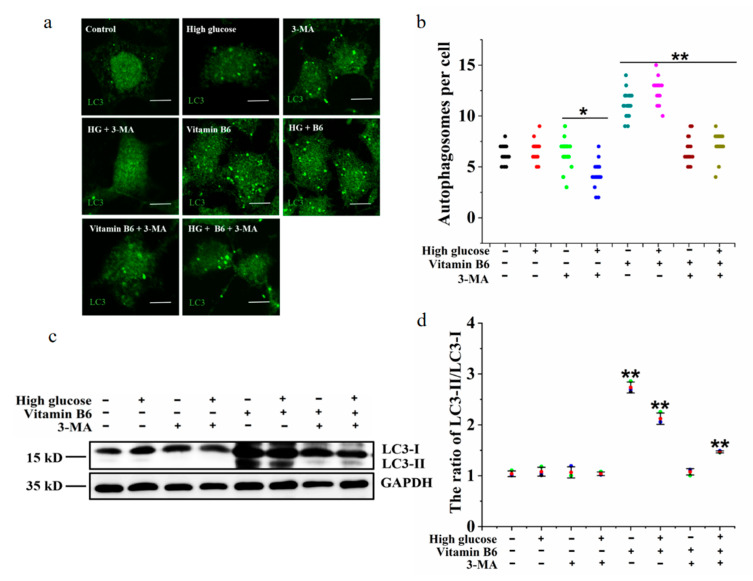
3-MA inhibited vitamin B6-induced autophagy under high glucose stress. (**a**) RIN-m5F cells were maintained with or without 20 mM glucose or with 10 mM 3-MA, 20 mM glucose plus 10 mM 3-MA, 20 μM vitamin B6, 20 mM glucose plus 20 μM vitamin B6, or 20 mM glucose plus 20 μM vitamin B6 plus 10 mM 3-MA, cultured for 36 h, and stained with anti-LC3 antibody (scale bar = 5 μm). (**b**) Cells were treated as described in (**a**), and the number of autophagosomes per cell was counted. At least 30 cells were analyzed. The experiment was repeated at least three times (Error bars, SD. ** *p* < 0.01, * *p* < 0.05 indicates significant differences compared with the control group by one-way ANOVA. (**c**) Cells were treated as described in (**a**), and Western blotting was performed to test the expression of proteins with specific antibodies against LC3 and GAPDH. (**d**) The LC3-II/LC3-I ratio in (**c**) was analyzed by IOD using Image-Pro Plus 6.0 software.

3-Methyladenine (3-MA) is an autophagy inhibitor. To test whether 3-MA can inhibit autophagy induced by vitamin B6 under high glucose stress, the number of autophagosomes, LC3-II/LC3-I ratio, protein expression of p62, and phosphorylation of p70S6K were tested after β-islet cells were treated with vitamin B6 or vitamin B6 plus 3-MA under high glucose stress. The results showed that the number of autophagosomes and the LC3-II/LC3-I ratio were increased significantly in the group treated with vitamin B6 plus high glucose (Figure 6a,b). However, the number of autophagosomes, LC3-II/LC3-I ratio, and level of phosphorylated p70S6K decreased after the cells were treated with vitamin B6 plus 3-MA and high glucose (Figure 6a,b). Thus, it can be concluded that 3-MA inhibits vitamin B6-induced autophagy under HG stress.

### 3.6. 3-MA Inhibits Vitamin B6-Induced Cytoprotective Autophagy against Apoptosis

**Figure 7:**
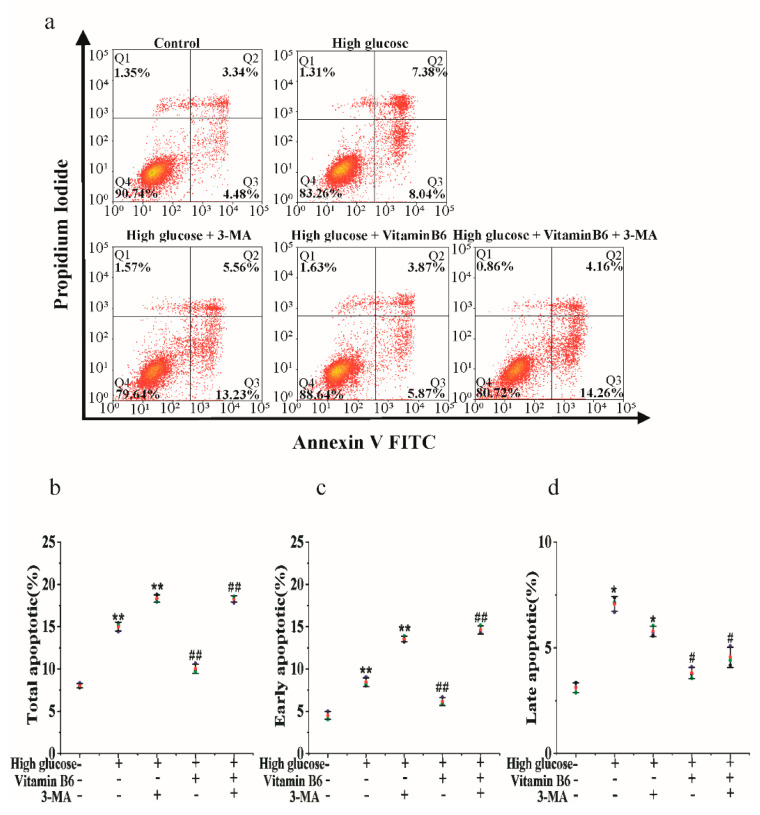
Vitamin B6 protected RIN-m5F cells from apoptosis by inducing autophagy under high glucose stress. (**a**) RIN-m5F cells were cultured with or without 20 mM glucose or with 20 mM glucose plus 10 mM 3-MA, 20 mM glucose plus 20 μM vitamin B6, or 20 mM glucose plus 10 mM 3-MA plus 20 μM vitamin B6, and the apoptosis rate was measured by flow cytometry. (**b**–**d**) Cells were treated as described in (**a**), and total apoptosis, late apoptosis, and early apoptosis rates were analyzed. The experiment was repeated at least three times (Error bars, SD.; ** *p* < 0.01, * *p* < 0.05 indicates significant differences compared with the control group by one-way ANOVA. ## *p* < 0.01, # *p* < 0.05 indicates significant differences compared to the vitamin B6 plus high glucose-treated group by one-way ANOVA.

To investigate the mechanism of the inhibition of high glucose-induced apoptosis by vitamin B6, cells were treated with vitamin B6 plus 3-MA under high glucose stress, and the apoptosis rate was tested by flow cytometry. Our data showed that vitamin B6 decreased the total apoptosis rate of islet β cells from 16.13% to 10.20%. 3-MA in combination with vitamin B6 increased the total apoptosis rate from 10.20% to 19.45% compared with the vitamin B6-treated group under high glucose stress (Figure 7a,d). These results suggest that vitamin B6 inhibited high glucose-induced RIN-m5F cell apoptosis, possibly via autophagy.

### 3.7. Effect of Hydrothermal Processing on the Biological Activity of Vitamin B6

**Figure 8:**
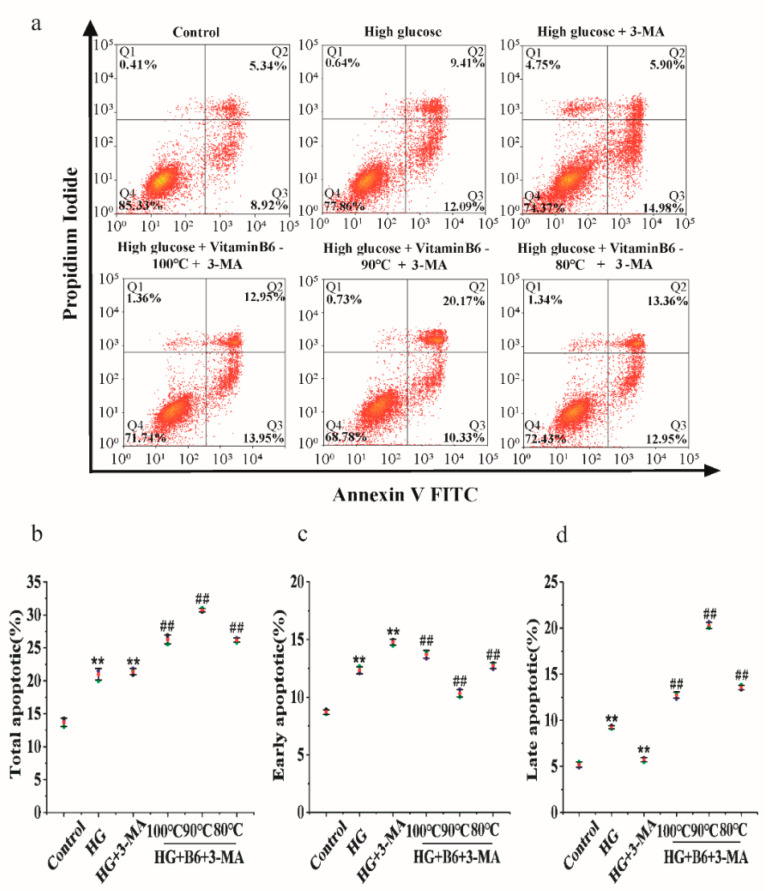
Hydrothermally processed vitamin B6 protected RIN-m5F cells from apoptosis by inducing autophagy under high glucose stress. (**a**) RIN-m5F cells were cultured with or without 20 mM glucose plus 10 mM 3-MA or with 20 mM glucose plus 20 μM hydrothermally processed vitamin B6, or 20 mM glucose plus 10 mM 3-MA plus 20 μM hydrothermally processed vitamin B6, and the apoptosis rate was measured by flow cytometry. (**b**–**d**) Cells were treated as described in (**a**), and apoptosis rates were analyzed (Error bars, SD ** *p* < 0.01 indicates significant differences compared with the control group by one-way ANOVA. ## *p* < 0.01 indicates significant differences compared with the control group by one-way ANOVA.).

To test the effect of hydrothermal processing on the biological activity of vitamin B6, vitamin B6 was heated in a water bath at 100 °C, 90 °C, or 80 °C for 15 min, and the apoptosis rate was measured by flow cytometry after cells were treated with the processed vitamin B6. Cells treated with hydrothermally processed vitamin B6 at all three temperatures had a decreased total apoptosis rate, while the total apoptosis rate increased from 20.88% in untreated cells to 26.90%, 30.50%, and 26.31% in cells treated with vitamin B6 heated to 100 °C, 90 °C, or 80 °C, respectively, in combination with 3-MA (Figure 8a–d). These data suggest that the hydrothermal process reduced the vitamin B6 content in the eggs but did not affect the biological activity of vitamin B6.

## 4. Discussion

Spinach, eggs, and meat are rich in vitamin B6. Eggs are an everyday food and one of the best sources of nutrition for humans [24]. It has been reported that dietary eggs increase the feeling of satiety by increasing fat oxidation and reducing the postprandial insulin response, plasma insulin, and carbohydrate oxidation [25,26,27]. Dietary eggs can also promote the feeling of satiety by preventing large deviations in blood glucose and insulin levels [25,26,27]. There are many processing methods for eggs. Hydrothermal processing is a convenient method to produce dried eggs [28,29,30]. However, the effects of hydrothermal processing on the content and biological activity of nutrients in eggs still need to be studied.

Vitamin B6 plays an important role in alleviating the complications of diabetes and preventing diabetes [31]. A deficiency of vitamin B6 leads to the occurrence and development of diabetes and its complications [4]. Vitamin B6 intake can also effectively reduce the occurrence of diabetes and its complications in pregnancy and obesity [32]. Currently, the treatment of diabetes mellitus is a difficult task for biomedicine throughout the world. Diet and exercise have been effective means to treat and alleviate diabetes [33]. A Mediterranean diet and effective sleep measures are effective means to prevent diabetes and its complications [34,35]. Intake of inositols and vitamin D, micronutrients, and pre/probiotics improved insulin sensitivity [36]. Intermittent fasting and a continuous energy-reduced diet were effective methods to reduce weight and hyperglycaemia [37]. A ketogenic diet (KD) is an effective approach to treat diabetes by regulating blood glucose and insulin levels [38]. Here, we found that vitamin B6 had a protective effect on islet cells by stimulating autophagy. Vitamin B6 is an essential nutrient. Its role in the remission and treatment of diabetes is gradually being confirmed. However, the molecular mechanism of the beneficial effects of vitamin B6 on diabetic pathology and its complications remain to be investigated.

Autophagy has been reported to regulate β-cell differentiation and promote survival under stressful conditions for β-cells, including nutrient deficiency, oxidative stress, ER stress, high glucose, and hypoxia [39]. It has also been shown to regulate the normal function of insulin target tissues, such as skeletal muscle, liver, and adipose tissue. Blood glucose-lowering drugs, such as metformin and SGLT2 inhibitors, reduced the risk of diabetic cardiovascular death, kidney disease, and heart failure by upregulating autophagy [40]. Drugs and natural products used to treat or alleviate diabetes can regulate autophagy through the mTOR, AMPK, ERK, and Hedgehog signaling pathways to exert a blood glucose-lowering effect [41,42]. The two mTOR complexes, mTORC1 and mTORC2, regulate cell growth, metabolism, and autophagy. mTORC1 regulates protein synthesis and autophagy by phosphorylating S6K and the protein kinase ULK1 [22], respectively. In this work, we showed that vitamin B6 induced autophagy by inhibiting the phosphorylation of p70S6K. Our data also suggested that vitamin B6 blocked the apoptosis rate of β-cells under high glucose stress. The autophagy inhibitor 3-MA blocked the cytoprotective activity of vitamin B6, so we concluded that autophagy induced by vitamin B6 inhibited apoptosis stimulated by high glucose. We also reported that hydrothermal processing decreased the content of vitamin B6 but had no effect on the biological activity of vitamin B6. Our data suggest that low temperatures and a short-term hydrothermal process are beneficial for dietary eggs because they can preserve the content and function of vitamins in eggs.

Hyperglycaemia is a clinical feature of diabetes. High glucose causes apoptosis of pancreatic islet cells, which eventually leads to impairment of β-cell function. High glucose promoted apoptosis by inducing oxidative stress [43,44]. The noncoding RNAs CA7-4, GCN2, gasdermin D, and miR-345-5p can also regulate high glucose-induced apoptosis [45,46,47,48]. In this paper, we found that high glucose induced β-cell apoptosis. Vitamin B6-induced autophagy inhibited β-cell apoptosis. We also found that high glucose increased reactive oxygen species (ROS) levels using a H2DCFDA (DCFH-DA) probe (Figure S1). However, the molecular mechanism by which high glucose induces apoptosis and the molecular mechanism by which vitamin B6-induced autophagy protects β-cells against apoptosis remains to be elucidated.

## 5. Conclusions

In this work, we showed that vitamin B6 induced autophagy under normal conditions and high glucose stress. Vitamin B6-induced autophagy inhibited apoptosis stimulated by high glucose via the mTOR-dependent pathway. Our data also suggested that the hydrothermal process reduced the vitamin B6 content in the eggs but did not affect the biological activity of vitamin B6.

## Data Availability

The data presented in this study are available in article and Supplementary Material here.

## Supplementary Materials

The following supporting information can be downloaded at: https://www.mdpi.com/article/10.3390/metabo12111048/s1, Figure S1: Glucose increased ROS levels in RIN-m5f cells.
