# Supplementary material for: Vitamin B6 Inhibits High Glucose-Induced Islet β Cell Apoptosis by Upregulating Autophagy
Source: Metabolites. 2022 Oct 31;12(11):1048. doi: 10.3390/metabo12111048 (PMC9695582; doi:10.3390/metabo12111048)
Supplement: Supplementary file 1 [file metabolites-12-01048-s001.zip › metabolites-1919395-supplementary.pdf]

a

b

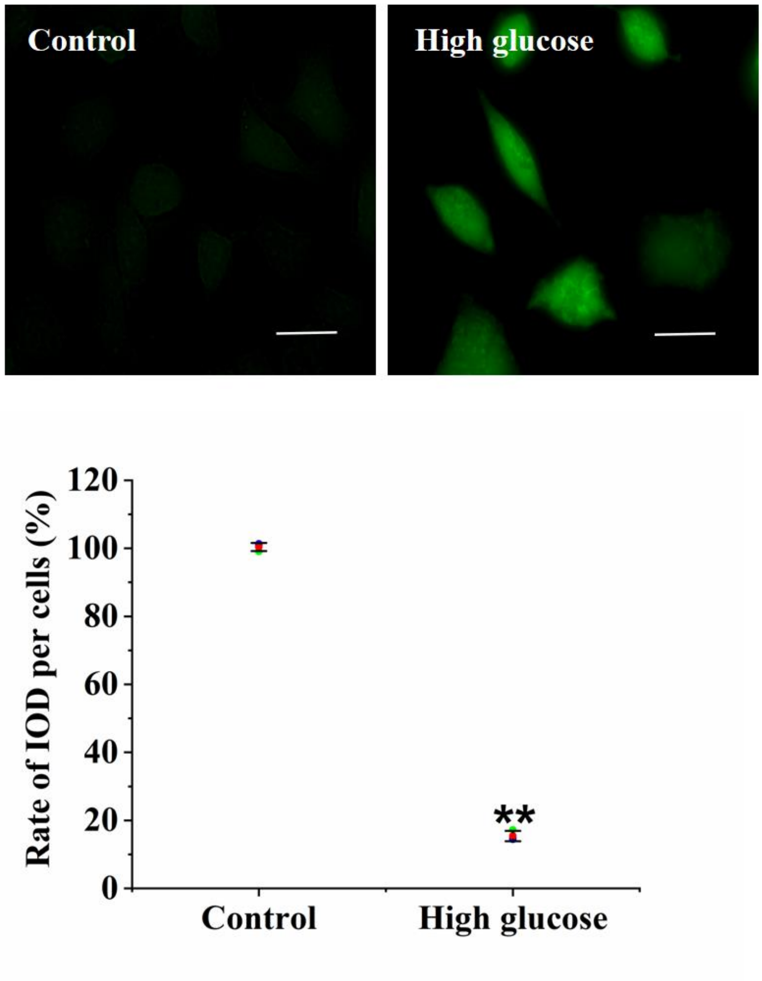

Figure S1. Glucose increased ROS levels in RIN-m5f cells . (a) The RIN-m5F cells were treated with or without 20 mM glucose, then the cells were incubated with DCFH-DA and fluorescence intensity was observed by fluorescence microscope. (b) Cells were treated as described in **a**, and fluorescence intensity were analyzed by the software Image-Pro-Plus 6.0 (Error bars, SD.;** *p* < 0.01 indicates significant differences compared with the control group by t test). Scale bar: 10 μm.
